# Forecasting the future of library and information science and its sub-fields

**DOI:** 10.1007/s11192-020-03800-2

**Published:** 2020-12-17

**Authors:** Zehra Taşkın

**Affiliations:** Scholarly Communication Research Group, Adam Mickiewicz University in Poznań, Poznań, Poland

**Keywords:** Forecasting, Library and information science, Sub-field analysis, Disciplinary differences, Time series analysis

## Abstract

Forecasting is one of the methods applied in many studies in the library and information science (LIS) field for numerous purposes, from making predictions of the next Nobel laureates to potential technological developments. This study sought to draw a picture for the future of the LIS field and its sub-fields by analysing 97 years of publication and citation patterns. The core Web of Science indexes were used as the data source, and 123,742 articles were examined in-depth for time series analysis. The social network analysis method was used for sub-field classification. The field was divided into four sub-fields: (1) librarianship and law librarianship, (2) health information in LIS, (3) scientometrics and information retrieval and (4) management and information systems. The results of the study show that the LIS sub-fields are completely different from each other in terms of their publication and citation patterns, and all the sub-fields have different dynamics. Furthermore, the number of publications, references and citations will increase significantly in the future. It is expected that more scholars will work together. The future subjects of the LIS field show astonishing diversity from fake news to predatory journals, open government, e-learning and electronic health records. However, the findings prove that publish or perish culture will shape the field. Therefore, it is important to go beyond numbers. It can only be achieved by understanding publication and citation patterns of the field and developing research policies accordingly.

## Introduction

Price (1963 p. 19, 1974, p. 166), predicted more than half a century ago that if the exponential growth of big science continued, we could have two scientists for each person and dog in the population in the future, and we could have one million academic journals by the 2000s. Today, an average of 2.3% of worldwide gross national product is devoted to research and development activities (World Bank 2018), and 8.5 out of every 1000 workers is employed as a researcher (Organisation for Economic Co-operation and Development 2020). The current total number of active journals published worldwide is 380,299 (*ULRICHSWEB Global Serials Directory* 2020), and at least the 73,299,923 articles have been published since Price published *Little Science, Big Science* in 1963.^1^ One of Price’s biggest concerns was that if the growth of big science continued in this way, there would be no scientist who would be able to read every paper (1974, p. 165). Even though we have not reached the number of journals estimated by Price, scientific outputs have still been increasing rapidly, and science is more difficult to follow than ever. In fact, the 90% of the research papers are never cited, and 50% of published research papers are never read by anyone else than the authors, reviewers and editors (Tripathy and Tripathy 2017, p. 198).

One of the most important problems caused by big science is the inequality of scientific practices in various fields. Big science requires large budgets, diverse research groups with numerous staff members and big laboratories. The high costs of big science create a continuous interplay between the status system, which depends on honour and esteem, and class (Merton 1968, p. 57). According to Allison and Stewart (1974, p. 599), several publications and citations are affected by this inequality. One of the problems that creates this inequality is disciplinary differences: authors’ productivity depends on their work discipline, popularity and experience (Allison 1980; Merton 1968). Even today, big science provides a cumulative advantage for some scientists and disciplines. This cumulative advantage, in turn, affects the distribution of science funds (Bol et al. 2018) and other scientific career decisions (Petersen and Penner 2014). Scientific rewards are much more unequally distributed than other well-being outcomes (Xie 2014, p. 810). For these reasons, the general characteristics of each discipline should be understood, and decisions should be made according to these characteristics to be able to make the right decisions in research evaluations.

Through the examination of the development of the LIS field, the same inequality can be seen. Over the years, studies have revealed that although the field is relatively small in the social sciences, it has several sub-fields, and the characteristics of these sub-fields are different from each other in terms of publication and citation patterns, authorship structures, production frequencies, etc. (Åström 2010; Moya-Anegón et al. 2006; White and McCain 1998). Besides, the development of sub-fields is directly affected by time and trends. For example, the number of articles written using terms such as ‘information technology’, ‘social network analysis’ or ‘citations’ has increased in recent years, but traditional librarianship topics such as librarianship, archiving or cataloguing have shown a decreasing trend (Larivière et al. 2012, pp. 1006–1009). While this can be advantageous for some sub-fields, it negatively affects the visibility of more traditional fields and causes an unequal distribution of funds and resources.

The main aim of this study is to determine the sub-fields of the LIS field, reveal the potentials of these fields and make predictions of each sub-field. This will highlight the different scientific practices within the same discipline, which must then be taken into consideration when making decisions. The research questions are as follows:

- What is the current structure of the LIS field and its sub-fields? Is there a significant difference between the sub-fields and publication/citation patterns?
- Based on a 10-year forecast using the publication information produced in the LIS field, what size increase might be expected in the number of future publications?
- Is it possible to predict the number of future citations? What are the citation potentials of the sub-fields?
- How will the number of references cited in LIS papers change in the future?
- Will the co-authorship patterns in the LIS field change in the future?
- Are the quantitative predictions consistent, and do they provide valid insights for the future?
- What are the emerging topics of the LIS field? Is it possible to predict future topics of LIS?

## Literature review

The literature review is organized into two main parts. The first part presents the subject distribution of papers published in the LIS field which use time series analyses. In the second part, various studies using time series analysis in scholarly communication and research evaluation fields are summarized. The explanation about the use of time series analysis is given in the Methodology section.

### Time series analysis studies in LIS

**Fig. 1:**
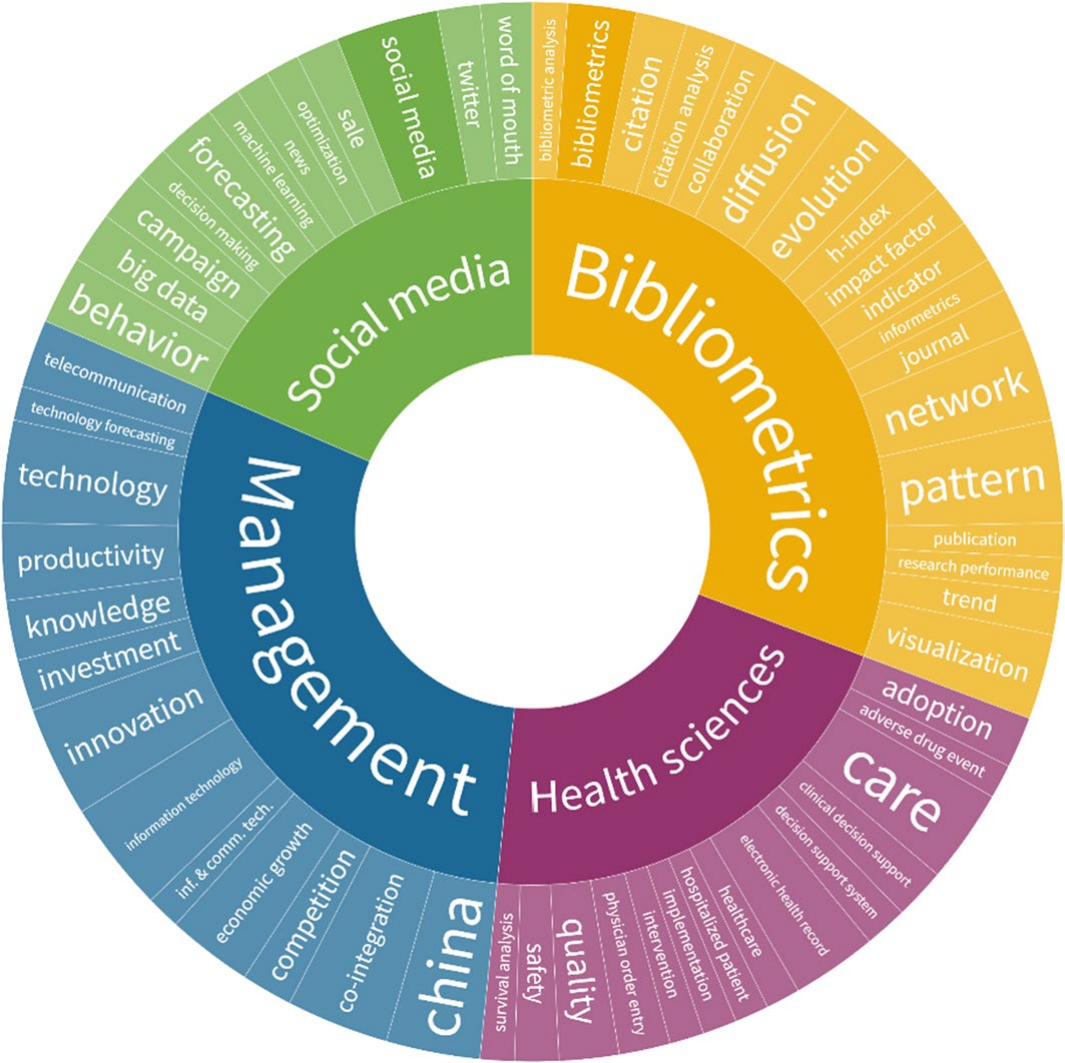
Most used keywords of the time series analysis studies in LIS (The sunburst graph was created by using Flourish Studio (https://app.flourish.studio/). Keyword occurrences were calculated by using VOSviewer. Before the calculation, the keyword standardization process was conducted.)

Time series analysis has been applied in the LIS literature to provide forecasts on four different sub-topics: Bibliometrics, health sciences, management and social media. To define main application areas of time series analysis in the field of LIS, 452 papers published in LIS and indexed in Web of Science were evaluated.^2^ (see Fig. 1).

An article by Bates et al. (1999) is the most cited paper with its 768 citations in the dataset, which includes publications indexed in Web of Science’s Information Science and Library Science category. The article evaluated the impact of computerised physician order entries to reduce the number of medication errors. The authors used prospective time series analysis to calculate the effectiveness of computerised systems for medications. As a result, it is found that computerised systems resulted in a large decrease in medication errors. The second-most cited paper (372 citations) evaluated time series data for online product reviews to understand the effects of word of mouth on online shopping (Li and Hitt 2008). The third-most cited article (283 citations) was written by the founder of CiteSpace and his colleagues (Chen et al. 2010). The authors used time series analysis to introduce a new multi-perspective co-citation analysis method for information science literature. The most-cited articles from three different sub-topics prove the subject diversity of publications which used time series analysis methods and techniques.

The bibliometric studies using time series analysis are focused on research evaluations, bibliometric indicators and scientometric visualisations. These studies have sought to reveal the relation between early citations and cumulative advantage (Adams 2005), evaluate the effectiveness of monetary support systems (Tonta 2018), understand the citation trajectories of Nobel prize winners in economics (Bjork et al. 2014), visualise or discover the intellectual structure of disciplines (Ma 2012) or events (Clausen and Wormell 2001), analyse the evolution of research topics (Wu et al. 2014), predict citation counts (Abrishami and Aliakbary 2019), observe the effects of science policy changes on the number of publications (Baskurt 2011), forecast research activities (Bildosola et al. 2017), detect emerging/leading papers (Iwami et al. 2014) and evaluate research metrics (Liu and Rousseau 2008; Ye and Rousseau 2008). The time series analysis techniques have been used in bibliometric studies since the early 1990s, and it is still one of the preferred methods in the literature. The main reason for this choice might be explained by the policymaking mission of research evaluations. Following the impact of research policy changes or detecting number of future citations provide important findings to the policymakers to enhance evaluation processes.

Time series analyses have also been used in the papers on health information. In recent years, the studies in health information have focused on evaluating electronic health records, predicting health risks (Perrote et al. 2015), optimising drug-drug interaction alert rules using electronic health records (Simpao et al. 2015), understanding information-seeking behaviours on health subjects (Huerta et al. 2016) and monitoring mental health discussions on Twitter (McClellan et al. 2017). The whole world has witnessed how long- and short-term predictions on health issues important during COVID-19 times. It is expected to see a publication explosion in this field in the future. Studies that make predictions on various issues related to the COVID-19 have started to be published in the literature (e.g. Jiang et al. 2020; Salgotra et al. 2020). Although there are many “unknown unknowns” exists about the virus, time series analysis is likely to be more popular among policymakers by providing a range of scenarios (Grogan 2020).

Economics and management sub-subjects of LIS field are also conducted research by using time series analysis. The papers have focused on telecommunication infrastructure and its relations to economic growth/activity (Cronin et al. 1991; Dutta 2001), disseminating economic census data (Zeisset 1998) and early detection of an economic bubble (Dmitriev et al. 2017). The last subject category, social media, can be accepted as a part of management subject. During the social media age, the predictions on big data (Niu et al. 2017; Saboo et al. 2016), social media analyses (Luo and Zhang 2013; Zhang et al. 2019), word of mouth (Li Hitt 2008) and election analyses on Twitter (Conway et al. 2015) are some of the important research topics.

The thematic diversity of LIS studies that have used time series analyses demonstrates that this is an essential method for scholars working in this field and is not limited to forecasting. In this study, the main aim of using a time series analysis was to make predictions about research outputs for the LIS field.

### Prediction types in the field of scholarly communication and bibliometrics

Forecasting the future is one of the most frequently discussed subjects in bibliometrics and research evaluation studies. Predictions are often made to estimate Nobel Prize Laureates by considering publication and citation patterns. The Web of Science group has provided this well-known prediction mechanism for Nobelists since 2002 (Bourke-Waite 2019). Since 1970, millions of indexed publications and citations to these papers have been evaluated and estimations made. Until 2019, 50 Nobel prize winners who were on the list of citation laureates won the Nobel Prize. Of these, 29 researchers received the prize within 2 years of being nominated. Besides the Web of Science Group, there have been other numerous papers published in the literature to predict Nobel Prize winners (e.g., Ashton and Oppenheim 1978; Claes and De Ceuster 2013; Siegel 2019); however, Gingras and Wallace (2010) warned against the limits of bibliometric tools for predicting Nobel Prize winners due to the rapid growth of disciplines and the halo effect.

Another important area of predictive research is estimating the future number of publications and citations using different tools, techniques and perspectives. Leydesdorff (1990) sought to estimate the national performance of EEC (European Economic Community) countries and the US using time series analysis models. He found that it is possible to predict the following year’s publication statistics. In Rousseau (1994) proposed a double exponential model for first citation processes. He aimed to find a model for first citations, and he suggested two models to predict the total number of articles in a fixed group that would ever be cited. In Burrell (2003) developed the theory of stochastic models to predict the future citation patterns of individual papers. He found that expected citation count was a linear function of the current number, thus proving the idiom ‘success breeds success’.

Chen (2012) proposed a theoretical and computational model to predict future citations using three metrics: modularity change rate, cluster linkage and centrality divergence. The results indicate that the model could successfully predict future citations. Also, authors’ collaboration statistics and the number of references were found to be good predictors of global citations.

From the citation perspective, Abbasi et al. (2011) created a model to identify the effects of co-authorship networks on scholars’ performance. As a result, they recommended using researchers’ networks to predict scholars’ future performance. Tahamtan et al. (2016) reviewed the literature and presented 28 factors affecting the number of citations, these factors were then sorted into three main categories: paper-related factors (such as quality of papers, document type, etc.), journal-related factors (such as the impact factor or journal’s language) and author-related factors. The authors indicated that it is possible to predict the frequency of citations by considering these factors. Similarly, Chakraborty et al. (2014) developed a two-stage prediction model that produced better results for highly cited papers, and the authors suggested using this model to predict seminal papers in the scientific fields. The authors indicated that although the publication’s authors and venue are crucial for gathering citations, the features related to the papers’ content are more effective for long-term citation predictions. Another study on estimating the factors affecting the number of citations received by articles published in 12 crime psychology journals showed that author impact might be a more powerful predictor of how many times an article is cited than the venue (journal) of publication (Walters 2006).

Brody et al. (2006) examined the relationship between the number of early downloads and the number of citations received for the publications on Arxiv. The study showed that there was a correlation between early downloads and citation impact. Besides, the longer the period for which downloads were counted, the higher the correlation between downloads and citation impact. The authors concluded that the 2 year citation impact should be estimated using 6 months of download statistics.

One of the most recent studies on citation data and forecasting investigated whether the number of volumes that the journals published affected the impact factors of the journals (Zhang 2020). The results showed that if the increase of volumes is consistent and significant, a decrease of impact factors is unlikely.

Unlike the other studies mentioned above, some of the studies in the literature did not aim to estimate the number of citations using different statistical data but rather to predict future technologies using citation data. Small (2006) proposed using clustering, mapping and string formation to track and predict growth areas in science. Érdi et al. (2013) developed a new model to detect new technological hot spots by clustering patent citation data. Similarly, the Bass and ARIMA models, which are time series analysis models, were utilised to forecast development trends based on patent data (You et al. 2017). Kwon and Geum (2020) indicated that promising inventions can be identified by considering the number of backward citations as the link with previous knowledge. All these studies demonstrate that time series analysis can not only be used to predict the number of outputs in the literature but also to forecast technological developments.

Considering the number of forecasting studies in the literature, predictions provide important findings for scholars, policymakers and managers working in LIS and its sub-fields. Through these findings, it is possible to develop policies, identify the problematic practices and measure the effects of policy changes.

## Methodology

### Data structure

To achieve the aims of the study, an advanced search of the Web of Science core indexes (SCI, SSCI and A&HCI) was conducted on 12 December 2019 using the search string *WC* = (*“Information science and Library Science”*) *AND LA* = (*English*) *AND PY* = (1921–2018) *AND DT* = (*article*). Although the Information Science and Library Science category is only indexed in SSCI, up to 5000 articles were indexed in SCI and A&HCI but not SSCI. Therefore, all three core indexes were included in the study to cover all studies in the field.

The oldest paper within the author’s subscription limits was from 1921, so that year became the starting point. Since the research was carried out before the end of 2019, the year 2019 was excluded from the scope of the research to avoid manipulation of the data and findings. However, the publication and citation data for 2019 were used to validate the success of the predictions made in this study. Also, only articles written in the English language were considered to avoid manipulation due to document type or language differences.

**Fig. 2:**
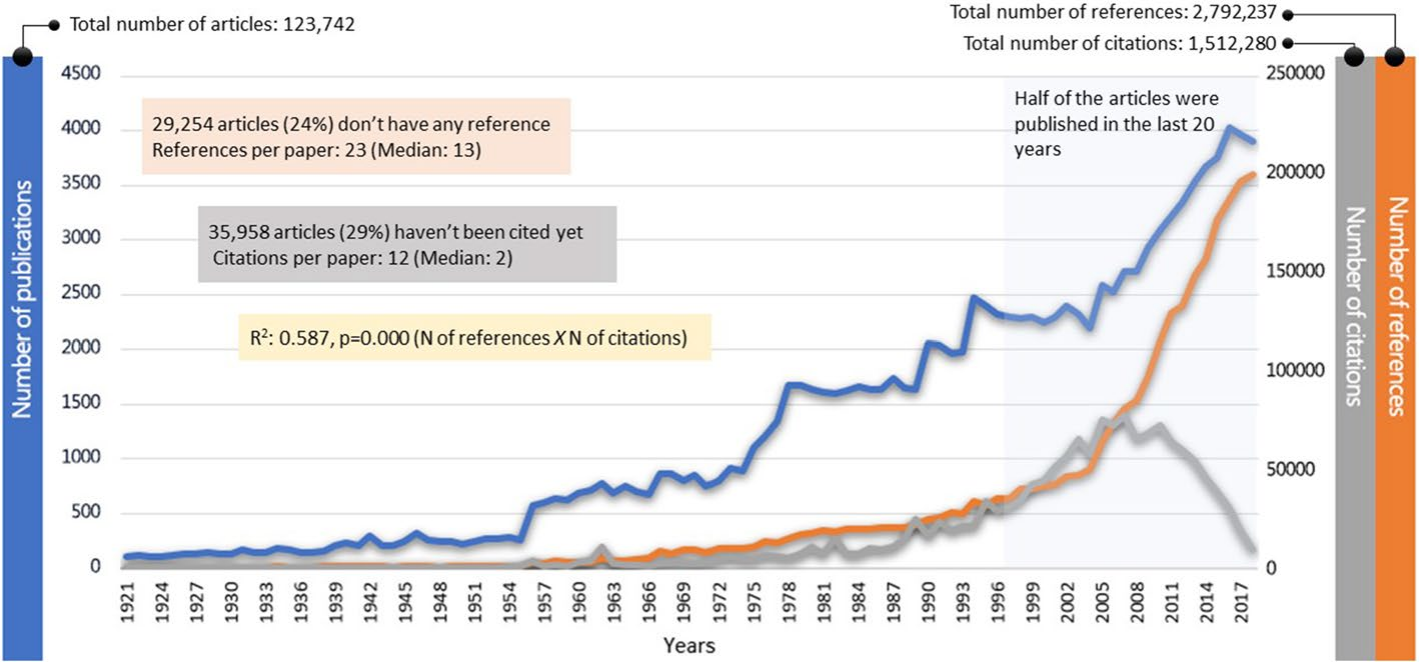
The main characteristics of the dataset.

A total of 123,742 articles were analysed and evaluated within the context of this study. The metadata of all articles was downloaded as tab-delimited text using the Web of Science exporting features. A total of 248 different .txt files were downloaded because of the download limits of Web of Science (500 records per download). Then, all the .txt files were combined using the command prompt.^3^ After creating one data file, a deep data cleaning and unification process was conducted. The main characteristics of the dataset are shown in Fig. 2.

The articles in the dataset were published in 174 different journals. To answer the research questions, the dataset was divided into four different sub-fields using social network analysis and clustering methods.

### Clustering and determination of LIS sub-fields

**Fig. 3:**
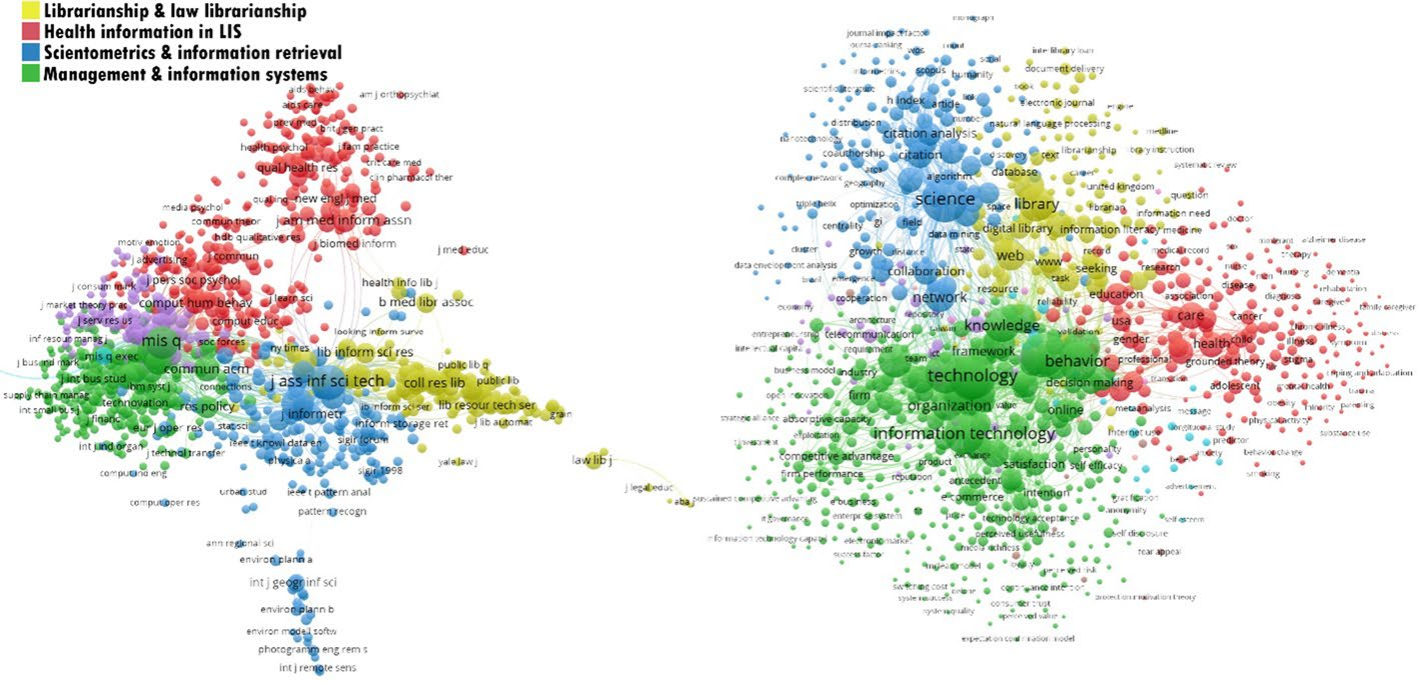
Clustering for journals in the dataset (networks of co-cited journals and keyword co-occurrence)

Two different networks were created for subject clustering. One was a co-cited journal network and the other was a co-occurrence of keywords network. The creation phases of the networks were:

- *Co-cited journals* The VOSviewer visualisation tool was used to create a co-citation network. Before creating the network, the names of the cited journals were standardised. During the standardisation process, different variations of journal names (e.g., Libr Trends, Lib Trends and Library Trends) and title changes (e.g., American Documentation, JASIS and JASIS&T) were considered. All journal names were unified. As a result, 537,227 sources were listed in our dataset. The limit for the minimum number of citations for a source was set at 20; 11,253 sources met this threshold. The co-citation network shown in Fig. 1 presents the top 1000 co-cited journals in the network.
- *Co-occurrence network* The same standardisation process was used to unify the terms that appeared in the title, abstract and keyword fields. The standardisation process included unification of singular/plural words, abbreviations, noun phrases and synonyms. All keywords and the full counting method were selected to create the co-occurrence network. A total of 71,389 keywords were determined, and 8123 of these appeared at least five times in the etwork. The first 1000 terms are shown in Fig. 3.

The main reason for creating two different network maps was to cross-validate the subject distribution of the dataset. Based on the clustering results, five clusters were determined for each network map. The clusters determined by most-occurred keywords were parallel with the co-cited journal network. It provided the opportunity to verify the accuracy of the classification. For both networks, the purple clusters were considered to be part of the green cluster. Therefore, the main subjects were classified into four main clusters for our study: *librarianship and law librarianship* (traditional library studies), *health information in LIS*, *scientometrics and information retrieval* and *management and information systems*.

Although some authors have argued that the journal citation reports (JCR) subject classification is problematic because it covers management information system (MIS) journals, which are different from other sub-fields (Larivière et al. 2012, p. 999; Ni and Sugimoto 2011), our classification results for this field align with previous studies in the literature (e.g., Moya-Anegón et al. 2006; Ni and Sugimoto 2011; Tseng and Tsay 2013) that the field is generally divided into four sub-fields: information science (including information retrieval and information seeking), library science (practical and research-oriented), MIS and scientometrics. In this study, we also added health information to these classifications.

**Fig. 4:**
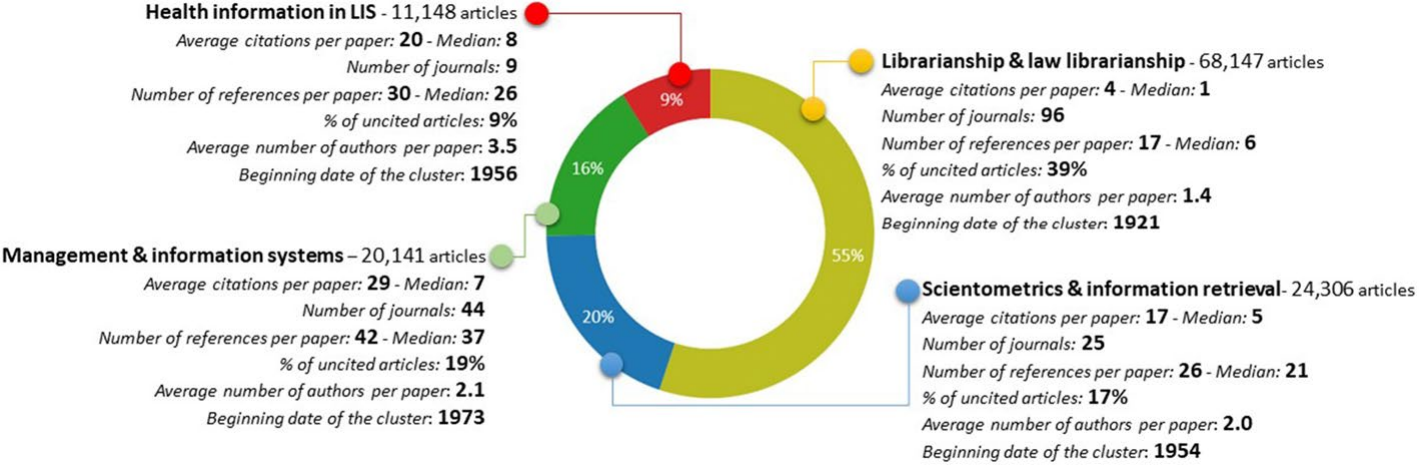
Distribution of journals into subject clusters

The main limitation of the classification used in this study was the journal-based approach. Some problems were determined for the journals which publish papers on two or more different topics. For example, the journal *Health Information and Libraries* was classified into the librarianship and law librarianship cluster by co-cited journal analysis, however, the main subject field of the journal is health libraries (Overview - Health Information and Libraries Journal 2020). To avoid that kind of problems, an expert control mechanism was conducted and content information from the articles published in that journal was used to decide the journal’s main focus. Additionally, if a journal was not listed in the network map, the same process was applied. For example, *African Journal of Library Archives and Information Science* was classified into the librarianship and law librarianship cluster using this method. The distribution of the articles into classes is shown in Fig. 4.

Each of the subject fields has different features even though they are all in the same subject category—LIS. Therefore, it is important to understand the structures of these sub-fields and their potentials. Although the librarianship and law librarianship category contains up to 50% of the articles, it is the field with the lowest citation rate. Furthermore, collaboration is more common for health information in the LIS literature. To understand the differences between the sub-fields, the Kruskal-Wallis test was conducted. The test showed that:

- The sub-categories of the articles significantly affect the number of publications that the articles cite (*H*[3] = 17951.379, *p* < 0.001),
- The sub-categories of the articles significantly affect the number of times an article is cited (*H*[3] = 19807.543, *p* < 0.001) and
- The sub-categories of the articles significantly affect the number of authors per paper (*H*[3] = 20557.826, *p* < 0.001).

The test results demonstrate that even if the study focused on a specific category, the sub-fields of that category could have different structures, and thus, evaluations must consider these differences.

### Time series analysis and time series forecasting

Many systems that we use today produce time-based data, which can be used to make various inferences. By using the data produced as a result of observations or experiments, problems with the system can be revealed, and predictions can be made about the future. The systematic approach to answering mathematical and statistical questions posed by time correlations is called *time series analysis* (Shumway and Stoffer 2006, p. 1). This method of analysis has been used in various fields, from economics to geographical sciences, and it has a wide range of applications. The literature review section summarized different variations of time series analyses in the LIS literature to achieve different aims.

Forecasting is one method of time series analysis and is used to provide the *t +* 1 value of future time by evaluating the *t* number of available observations (Box et al. 2008, p. 2). The forecasting process includes seven phases: (1) problem definition, (2) data collection, (3) data analysis, (4) model selection and fitting, (5) model validation, (6) forecasting and model deployment and (7) monitoring forecasting model performance (Montgomery et al. 2008, p. 12). SPSS Statistics 23 (IBM) was used to conduct the model selection, fitting, validation, deployment and monitoring phases of this study.

There are different types of time series data, and this must be considered when choosing the analysis method. The well-known data types in time series analyses are trend data, seasonal data and cyclical variations. As seen in Fig. 2, our dataset shows a linear trend, and thus the analyses were conducted to predict the future of this trend. The only exception for our data was the citations. Any publication requires a certain period to gather citations, and this period varies from discipline to discipline. The decrease in the number of citations over the last 8 years (Fig. 2) indicates that the half-life of citations in the LIS field is 8.3 (Incites Journal Citation Reports 2018). To prevent this decline from adversely affecting the results of the forecasting, only citation data up to 2010 were used. Thus, time series forecasting was applied using the period from 1921 to 2010.

Unusual events, disturbances or errors that might affect time series data are known as outliers (Box et al. 2008, p. 536). There are different methods to remove outliers from the data or to normalise the data to provide strong predictions. Removing or normalising citation data was vital for this study because there were too many extreme values, and without processing the data to remove outliers, it would have been impossible to provide a powerful forecast for research outputs in the LIS field. To achieve this aim, median scores of the number of references and the number of citations per year were used to normalise the data. Additionally, autocorrelation and partial autocorrelation plots were created (Appendix).

## Findings

The results of the forecasting analyses are presented in this section according to the number of publications, number of citations, number of references and number of authors per title.

### Number of publications

**Fig. 5:**
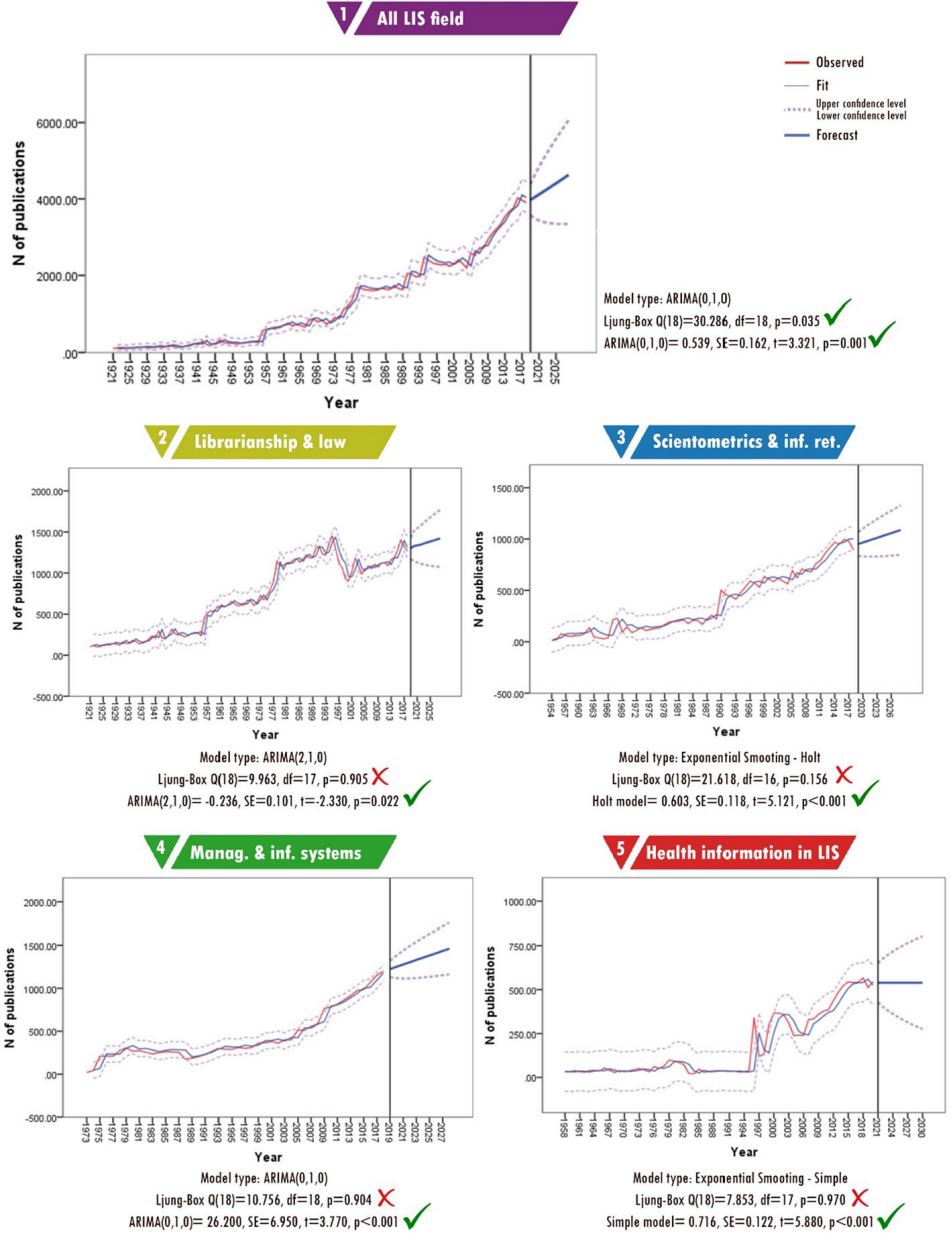
Forecasting for number of publications

As shown in Fig. 5, it is predicted that the number of publications in the LIS field will increase in the future. The average number of English language articles published per year in these 97 years was 1262; however, 50% of these articles were published in the last 20 years.^4^ Thus, an increasing publication pattern can be easily seen in Graph 1 in Fig. 5 (all LIS fields). Forecasting the number of publications for the whole LIS literature produced significant results [*Ljung Box Q*(18) *= 30.286, df* = 18, *p* = 0.035, *ARIMA*(0, 1, 0) = 0.539, *SE* = 0.162, *p* = 0.001], and according to the results, 3974 publications were predicted for 2019 and 4632 for 2027.

Because the expected number of articles for 2019 was estimated at 3974, and most of the articles published in 2019 are indexed in the Web of Science, it was possible to compare the forecast to the actual number of publications in 2019. A total of 4412 English language articles were published in 2019 and indexed before October 2020.^5^ This shows that the number of publications will likely increase beyond the prediction, as that many articles are not expected until 2024 in the time series analysis. However, this number is still within the limits of the upper confidence level. If we follow the upper confidence level of the forecast to estimate the near future, there may be 6069 published articles in 2028. This means that if the upper confidence levels are actualised, a total of 52,807 articles could be published between 2019 and 2028.

Although the forecasting tests for sub-fields produced meaningful results, the data were not sufficient to make predictions. It is possible to follow the data from the trend lines and Ljung-box scores. Results of the analysis suggest that increases are expected in the number of publications that will be produced in all sub-fields. This is evidenced by the fact that the forecasts and the actual numbers are quite similar (see Table 1), indicating that estimating the number of publications in the LIS field and its sub-fields is possible using time series analysis.

**Table 1:** Expected and actual number of publications in 2019

Sub-field	2019 actual	2019 forecast	Upper confidence level	Lower confidence level
Librarianship and law librarianship	1517	1300	1430	1170
Management and information systems	1324	1225	1319	1131
Scientometrics and information retrieval	997	951	1067	834
Health information in LIS	574	539	651	427

### Number of citations

**Fig. 6:**
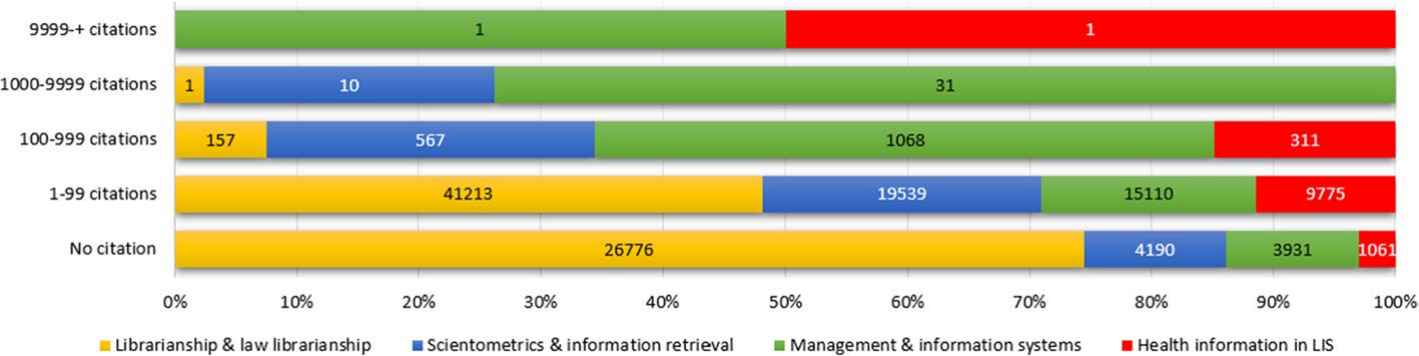
Distribution of citations according to sub-fields

Approximately 17% of the articles published in the LIS field have received 80% (1,209,824) of the citations for the whole literature. These statistics are important in terms of showing the existence of core articles in the LIS field. It is important to note that some publications receive numerous citations while others do not. Fig. 6 shows the distribution of citations received by sub-fields.

Analysis of the dataset shows that two articles received 10,000 citations. These articles were classified into the sub-fields of management and information systems and health information in LIS. The citation potentials are different for each category. For example, papers published in the sub-field of management and information systems are more likely to be cited than those published in the sub-field of librarianship and law librarianship. One of the main features of citation data is their skewness (Bornmann and Leydesdorff 2017), and my dataset was no exception. This skewness makes it difficult to produce accurate forecasts for the number of citations in the future.

In addition to the skewness of citation data, other problems are literature obsolescence and citation half-lives. Since the cited half-life of the LIS field is 8 years, it is not possible to make an accurate prediction using data from the last 8 years. For this reason, forecasting only covered the years 1921–2010, and the last 8 years were excluded.

**Fig. 7:**
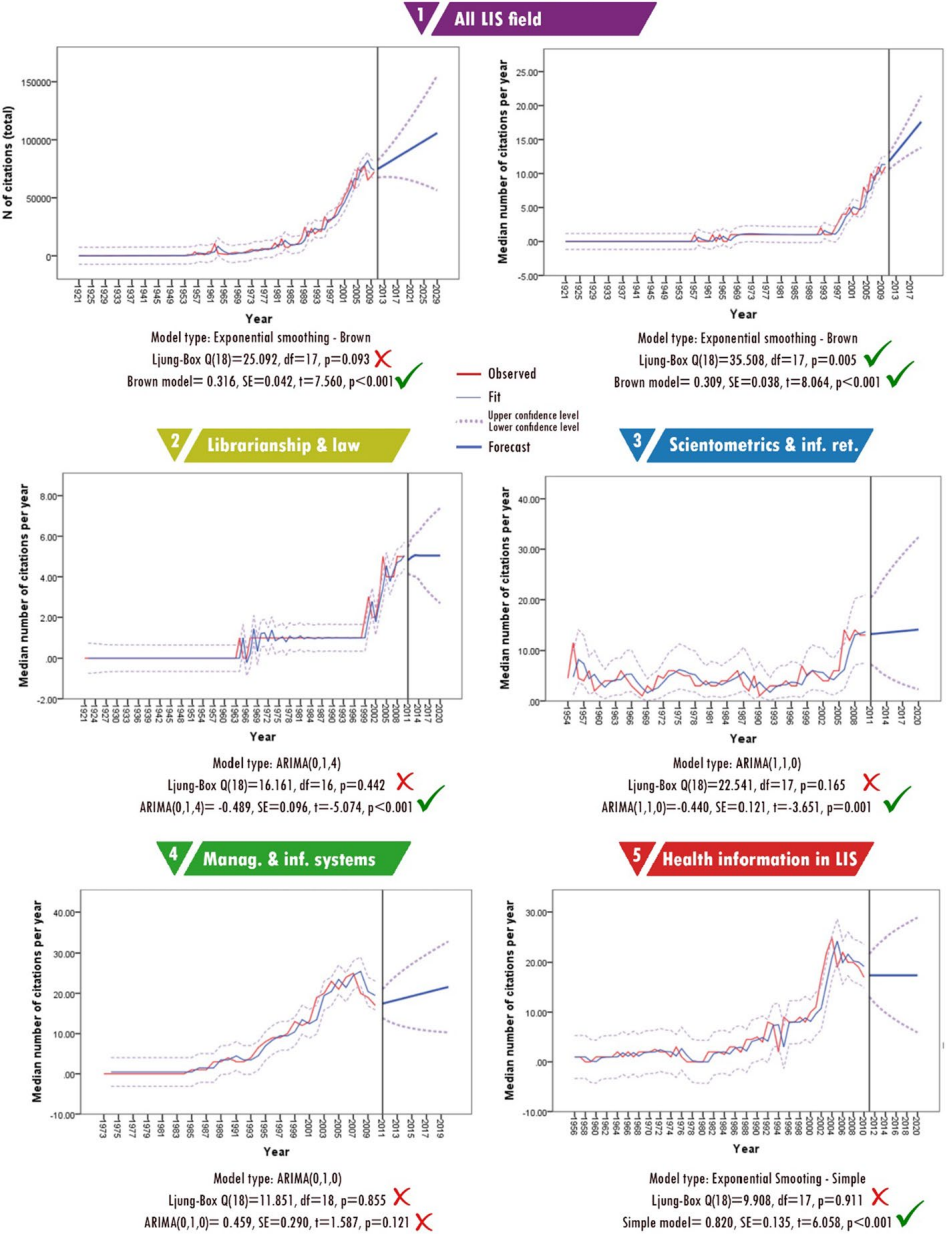
Forecasting for citations

Fig. 7 presents the forecasting results which show that the most consistent prediction could be obtained by analysing the entire discipline. However, for the field-based analyses, the predictions did not produce meaningful results. The results indicate that half of the publications could be cited 20 or more times per year in the future. Considering that the median number of citations currently is 10 per year, this prediction of a major increase in citation counts is possible. However, it is estimated that the number of citations received in the LIS field might exceed 100,000. If we assume the same upper confidence level as we did for the number of publications, the upper confidence for the total number of citations is estimated to be 141,000. Since the distribution of median values does not offer a linear trend for sub-fields, it is difficult to predict which sub-fields will receive more citations. Furthermore, the half-life may be different for each sub-field. This is one of the factors that makes forecasting difficult. Considering all these factors, future analyses might produce more meaningful results.

### Number of references per paper

**Fig. 8:**
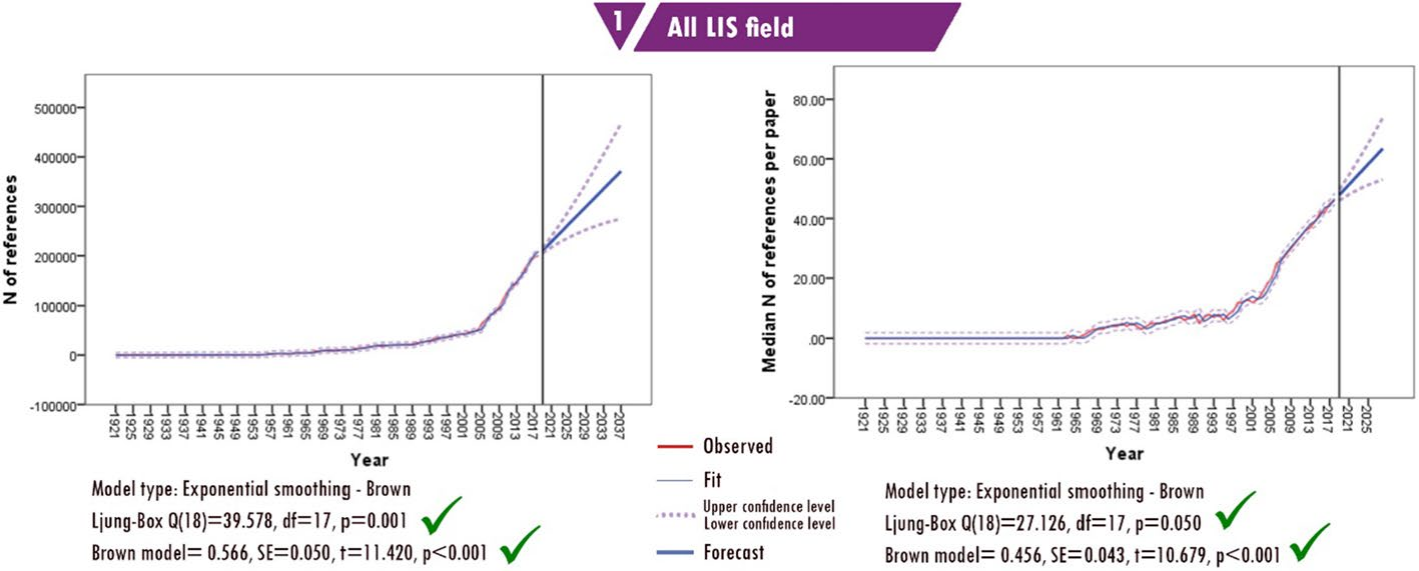
Forecasting of the number of references

While the number of references that could be cited in publications was more limited in the past, with the increase in the number of publications, there has also been a significant increase in the number of references made in studies. It is possible to monitor this increase from the trendlines in Fig. 8. The forecast predicts that a total of 300,000 references will be listed in the LIS literature in 2028. Half of the publications are expected to cite at least 63 sources. In 2018, this number was 47. The tests for forecasting produced significant results, and upper and lower confidence level scores were very close, indicating the accuracy/consistency of the future prediction.

Despite the success in forecasting the future number of references, it is difficult to make a similar forecast for the sub-fields because of the differences between the fields and the skewness of the reference/citation data. Although they are in the same main subject category, the citation patterns are different for each sub-field. The average number of references per article is 15 (median = 21) in the field of librarianship and law librarianship, 26 (median = 21) in scientometrics and information retrieval, 30 (median = 26) in health information in LIS and 42 (median = 37) in the management and information systems fields.

### Forecasting author collaborations

The average number of authors per paper in the LIS literature is two, and the median is one. Thus, scholars in the LIS field generally prefer to work alone. However, health information in LIS is the most collaborative sub-field of LIS literature. The article entitled ‘Academic domains as political battlegrounds: A global enquiry by 99 academics in the fields of education and technology’^6^. is the most collaborative paper with 99 authors. The article is classified as part of the librarianship and law librarianship sub-field in our dataset. The main statistics for authorship patterns are shown in Table 2.

**Table 2:** Co-authorship patterns of LIS field

Field or sub-field	Median author N	Average author N	Max author N
Entire LIS field	1	1.9	99
Librarianship and law librarianship	1	1.4	99
Scientometrics and information retrieval	2	2.0	23
Management and information systems	2	2.1	22
Health information in LIS	3	3.5	36

**Fig. 9:**
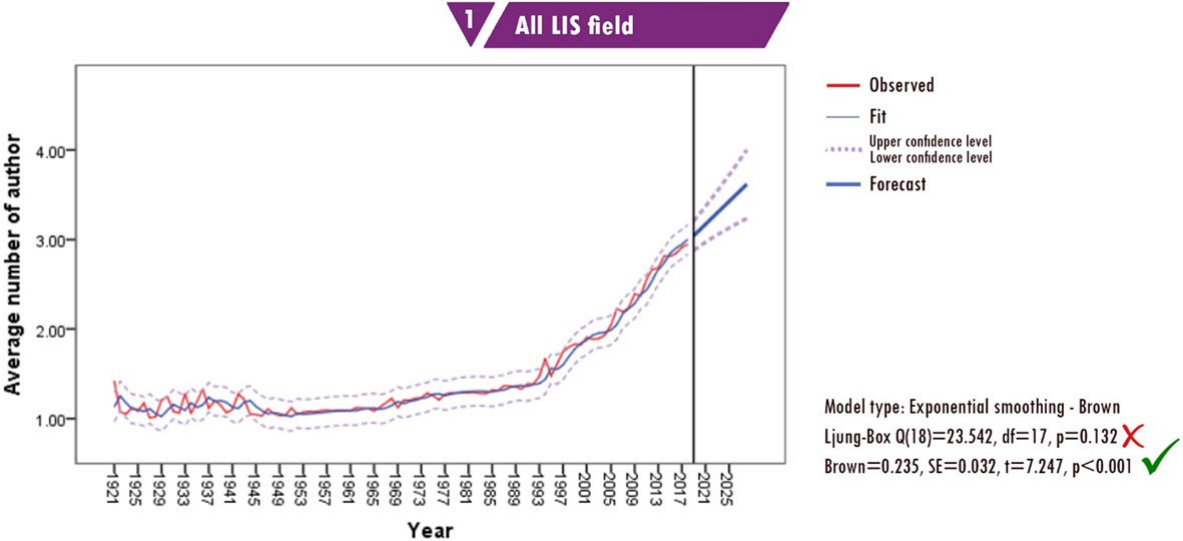
Forecasting of collaboration patterns

The forecast results show that the average number of authors per paper is three. In the next 10 years, this number is expected to increase to 3.6. Considering the trendline over the past 97 years, this expected result is reasonable. Fig. 9 presents the forecasts for collaboration patterns in the LIS literature.

### Possibility of consistent forecasting in the publish or perish world

**Fig. 10:**
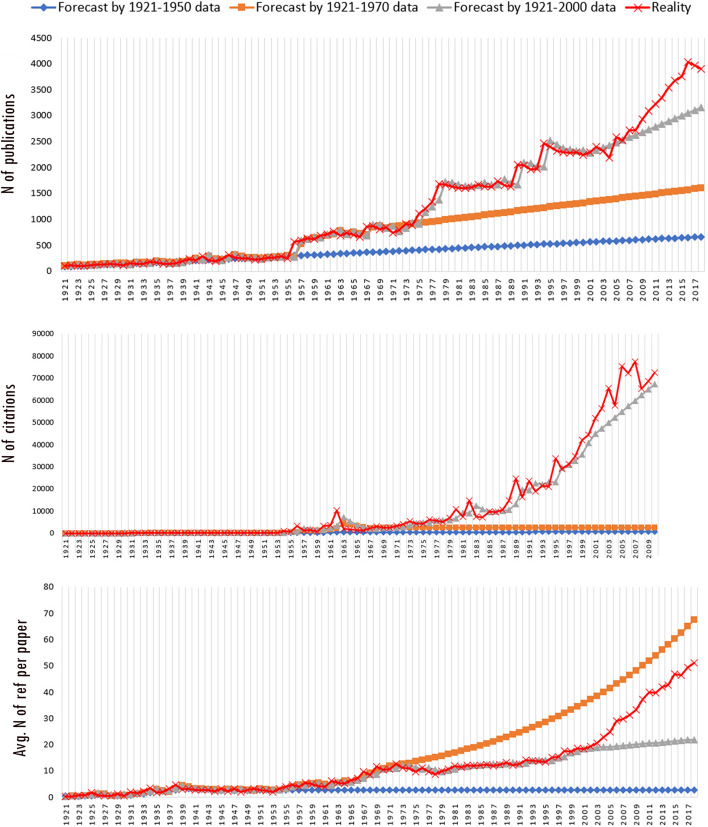
Forecasts by different periods

The analyses above demonstrate the difficulty of predicting research outputs. Every year, the number of publications increases. Since no regular trend can be seen in this increase in the number of publications, any predictions we make today are minimum values for the future. Table 1 presents one example of this. All three graphs in Fig. 10 show the estimated increase per year in comparison with the previous period. The periods are determined by considering the years that had increases in the number of publications.

Figure 10 demonstrates that the number of publications does not have a regular trend. Thus, there is the possibility that no prediction will accurately forecast the number of future publications. If the trend until 1950 had continued to today, the number of publications in the LIS literature today would be 37,049 (30% of today’s actual number). If the data from 1970 were used, the number would be 76,612 (61% of today’s actual number), and if the data from 2000 were used, the number would be 117,336 (94% of today’s actual number). While it is possible to say that forecasts in recent years have been more accurate, that is, the publication trends have been similar in recent years, the unpredictability should be expected to continue regardless of any changes in research performance evaluation systems. Besides, it should be kept in mind that the number of publications may be indirectly affected by unexpected emerging issues such as COVID-19 that significantly affect the publishing patterns of the authors.

It is difficult to estimate the total number of citations using the data up to 1970 because of the cumulative nature of citations. However, predictions using data up to 2000 produced forecasts that are close to reality. Using data up to 1970, it was estimated that the average number of references per paper would be 67 in 2018. However, the data after 1970 changed the situation. Using the data until 2000, the estimated average number of references per paper in 2018 was 22, while the actual average number of references in 2018 was 51. Thus, the number of references in publications have increased far beyond the predictions made using more recent data.

### Forecasting the research subjects

**Fig. 11:**
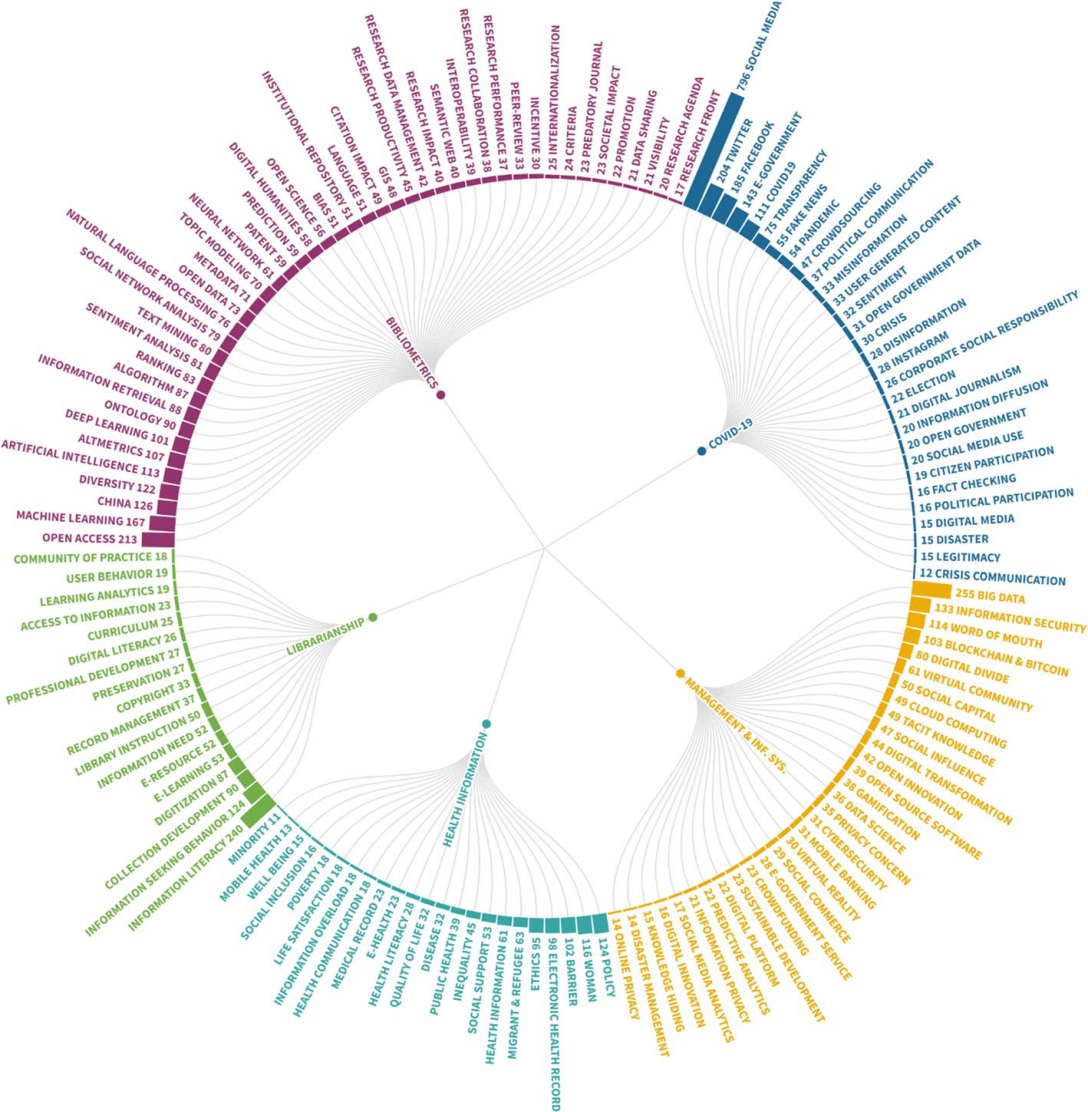
Emerging subjects of the LIS field (Flourish Studio was used to create the radial tree)

The findings confirm that the entire LIS field will face many more publications in the future with the spread of publish or perish culture. Therefore, the key to following the developments in LIS is to go beyond numbers. Although it is difficult to forecast the potential future of LIS subjects by using just numbers, making inferences by looking at the emerging subjects in recent years is possible. Figure 11 shows the most-used keywords of the papers published in the last two years in the LIS field.^7^

Figure 11 shows a network of keywords that includes five clusters. The four clusters are parallel with the classification of this study. However, a new cluster named “COVID-19” has been added to the LIS literature as expected. The emerging subjects of each sub-field are:

- *COVID-19* All the countries have been fighting with COVID-19 since December 2019. According to WHO’s COVID-19 Global Research Database (*Global Research on Coronavirus Disease *(*COVID-*19) 2020) a total of 123,959 publications were published from the day of the outbreak to November 7, 2020. The subject is also popular in the LIS field. Social media, fact-checking, governmental responsibilities during the pandemic such as political communication, transparency and participation, digital journalism and fake news are the important subjects of LIS field recently. The cluster proves the importance of LIS research focusing on open and correct information all over the world during the pandemic.
- *Bibliometrics and information retrieval* The keywords of this cluster show that bibliometrics and information retrieval issues converge to each other with the developments of computational techniques. Machine learning, text mining, topic modelling and sentiment analyses are used for bibliometric studies such as content-based citation analyses and digital humanities. Also, scholarly communication subjects like peer-review, societal impact, incentives, predatory journals, language (multilingualism) and rankings are important keywords of this cluster. As indicated in the literature review part, predictions are still important for this sub-field.
- *Librarianship and law librarianship* The effects of COVID-19 is also observed in this cluster (e.g. e-learning, e-resources). Information literacy plays a vital role among researchers, students and the public during COVID-19 times. Therefore, traditional librarianship subjects will be important to solve information problems of individuals in the future. Besides, digitization and preservation of archival materials are the other popular subjects of the cluster.
- *Health information in LIS* Many studies in this sub-field focus on disadvantaged groups in recent years. Studies on inequality, refugees and genders can be evaluated in this content. Also, public access to health information, health communication and electronic health records are popular subjects and related to COVID-19 pandemic.

## Discussion and conclusion

The study suggested a forecasting mechanism for research outputs in the LIS field. The main aim of the study was to inform scholars and policymakers about the future of research in this field. Nowadays, articles are often only read by a few people (Eveleth 2014; Simkin and Roychowdhury 2015; Tripathy and Tripathy 2017), and the main purpose of publishing is to achieve a numerical advantage rather than further the development of science. Although many researchers have emphasised that the current system should change, there have not yet been any concrete changes.

First, we revealed that publishing, citation and collaboration patterns differ between the sub-fields in the LIS literature. It is a well-known fact that apples and oranges are incomparable in research evaluations (Johnes and Johnes 1992); however, this study shows that it is also difficult to compare apples to each other because there are different types of apples (e.g., red, granny smith, honeycrisp, etc.). According to the results of the study more articles are published in traditional librarianship journals, and these journals tend to be cited less than others in the field. Articles published in the management dimension of the LIS field have greater citation potential than other sub-fields. This explains why management journals tend to have the highest impact factor in JCR among the LIS journals. This study shows the sub-field differences in LIS, and any evaluations based on categories should consider the sub-fields and their different characteristics.

The findings of this study indicate the number of publications and citations will continue to increase each year unless there is a change in research evaluation systems. This could lead to an uncontrollable mass of publications in the LIS field. The upper confidence levels estimated by the forecasting model produced in this study were already realised in 2019, demonstrating that this increase will be huge. However, it is difficult to forecast the future of sub-fields because the publication trends in sub-fields differ greatly from the general framework. If the existing systems continue, the inequality between LIS sub-fields will continue to grow. The meaning of following the current research evaluation systems is that the production of papers will continue to increase, and some of the sub-fields will not be able to benefit from future opportunities due to their disadvantages. For this reason, decision-makers and managers must consider field- and time-based differences in their research evaluation tasks.

One of the important results revealed in this study is the predicted increase in the number of publications, citations and references. Given that evaluations are made using citation data, the growing amount of data will make future evaluations more difficult. For this reason, supporting programmes such as the Initiative for Open Citations, which aims to promote the unrestricted availability of scholarly citation data, may also be useful for managing data in the future.

The results show that a lot of papers which have long reference lists will be produced, they will cite each other, more authors will work together to write papers. However, their contents and levels will be different from each other. Many of the studies have predicted that publishing will change in the future as a consequence of these differences. For example, Priem (2013) claimed that publishing forms, reward systems, measurement tools and peer-review systems will soon change. Similarly, Waldrop (2008) and Kendall (2015) stated that open science will be the new norm and that we will experience many changes to authorship and research evaluation systems in the next years. The predictions for the future of the publishing system is also the subject of the LIS field. This study proves the astonishing diversity of research subjects of the LIS field and tries to show the importance of looking beyond numbers.

## Appendix

Residual autocorrelation function (ACF) and residual partial autocorrelation function (PACF) plots

## Footnotes

1 The search was conducted on 7 July 2020 using the term *PY* = (1963–2020) in Web of Science’s core indexes: social sciences citation index (SCI), social sciences citation index (SSCI), Arts and humanities citation index (A&HCI), Emerging sources citation index (ESCI), Conference proceedings citation index (CPCI) and Book citation index (BKCI).

2 To access papers on/using time series analyses, the search string TS=(“time series” OR “forecasting analys*s” OR ARIMA OR “Exponential smoothing”) AND WC=(“Information science & library science”) was used. The search was conducted on 15 May 2020 using the Web of Science core indexes.

3 The text files were combined into a single file using the following steps: (1) open command prompt, (2) enter the folder, (3) use the code >> *copy *.txt join.txt* .

4 Although half of the articles were published in the last 20 years, using the entire past provides advantages for time series analyses using linear data (Jones 1964, p. 47). The rate of increase in the number of publications over the years is one of the important factors for the success of the forecast. To provide accuracy on forecasts, the entire 97-year period was used for prediction.

5 A total of 137 articles were identified as early access. These articles might be covered by volumes/issues published in 2020. The total number of articles excluding early access articles was 4275.

6 https://journals.sagepub.com/doi/full/10.1177/0266666916646415.

7 An advanced search was conducted on 5 November 2020 using the search string *WC=*(*“Information Science and Library Science”*) *AND PY* = (2019–2020) to gather the publication data for the last two years. All Web of Science indexes including ESCI, CPCI and BKCI were included to be able to cover more papers. Articles, reviews and proceedings were considered. A total of 13,856 papers were evaluated to analyze the emerging subjects of the field.

## References

[CR1] Abbasi A, Altmann J, Hossain L (2011). Identifying the effects of co-authorship networks on the performance of scholars: A correlation and regression analysis of performance measures and social network analysis measures. Journal of Informetrics.

[CR2] Abrishami A, Aliakbary S (2019). Predicting citation counts based on deep neural network learning techniques. Journal of Informetrics.

[CR3] Adams J (2005). Early citation counts correlate with accumulated impact. Scientometrics.

[CR4] Allison PD (1980). Inequality and scientific productivity. Social Studies of Science.

[CR5] Allison Paul D, Stewart JA (1974). Productivity differences among scientists: Evidence for accumulative advantage. American Sociological Review.

[CR6] Ashton SV, Oppenheim C (1978). A method of predicting Nobel Prizewinners in chemistry. Social Studies of Science.

[CR7] Åström F (2010). The visibility of information science and library science research in bibliometric mapping of the LIS Field. Library Quarterly.

[CR8] Baskurt OK (2011). Time series analysis of publication counts of a university: What are the implications?. Scientometrics.

[CR9] Bates DW, Teich JM, Lee J, Seger D, Kuperman GJ, Ma’luf N, Boyle D, Leape L (1999). The impact of computerized physician order entry on medication error prevention. Journal of the American Medical Informatics Association.

[CR10] Bildosola I, Gonzalez P, Moral P (2017). An approach for modelling and forecasting research activity related to an emerging technology. Scientometrics.

[CR11] Bjork S, Offer A, Söderberg G (2014). Time series citation data: The Nobel Prize in economics. Scientometrics.

[CR12] Bol T, de Vaan M, van de Rijt A (2018). The Matthew effect in science funding. Proceedings of the National Academy of Sciences.

[CR13] Bornmann L, Leydesdorff L (2017). Skewness of citation impact data and covariates of citation distributions: A large-scale empirical analysis based on Web of Science data. Journal of Informetrics.

[CR14] Bourke-Waite, A. (2019, September 24). The Web of Science Group reveals annual citation laureates of ‘Nobel class’. https://clarivate.com/news/the-web-of-science-group-reveals-annual-citation-laureates-of-nobel-class/

[CR15] Box GEP, Jenkins GM, Reinsel GC (2008). Time series analysis: Forecasting and control.

[CR16] Brody T, Harnad S, Carr L (2006). Earlier web usage statistics as predictors of later citation impact. Journal of the American Society for Information Science and Technology.

[CR17] Burrell QL (2003). Predicting future citation behavior. Journal of the American Society for Information Science and Technology.

[CR18] Chakraborty T, Kumar S, Goyal P, Ganguly N, Mukherjee A (2014). Towards a stratified learning approach to predict future citation counts. IEEE/ACM Joint Conference on Digital Libraries.

[CR19] Chen C (2012). Predictive effects of structural variation on citation counts. Journal of the American Society for Information Science and Technology.

[CR20] Chen C, Ibekwe-SanJuan F, Hou J (2010). The structure and dynamics of cocitation clusters: A multiple-perspective cocitation analysis. Journal of the American Society for Information Science and Technology.

[CR21] Claes AGP, De Ceuster MJK (2013). Estimating the economics Nobel Prize laureates’ achievement from their fame. Applied Economics Letters.

[CR22] Clausen H, Wormell I (2001). A bibliometric analysis of IOLIM conferences 1977–1999. Journal of Information Science.

[CR23] Conway BA, Kenski K, Wang D (2015). The rise of Twitter in the political campaign: Searching for intermedia agenda-setting effects in the presidential primary. Journal of Computer-Mediated Communication.

[CR24] Cronin FJ, Parker BP, Colleran EK, Gold MA (1991). Telecommunications infrastructure and economic growth: An analysis of causality. Telecommunications Policy.

[CR25] Dmitriev A, Dmitriev V, Sagaydak O, Tsukanova O (2017). The application of stochastic bifurcation theory to the early detection of economic bubbles. Procedia Computer Science.

[CR26] Dutta A (2001). Telecommunications and economic activity: An analysis of granger causality. Journal of Management Information Systems.

[CR27] Érdi P, Makovi K, Somogyvári Z, Strandburg K, Tobochnik J, Volf P, Zalányi L (2013). Prediction of emerging technologies based on analysis of the US patent citation network. Scientometrics.

[CR28] Eveleth, R. (2014, March 24). Academics write papers arguing over how many people read (and cite) their papers. *Smithsonian Magazine*. https://www.smithsonianmag.com/smart-news/half-academic-studies-are-never-read-more-three-people-180950222/?no-ist

[CR29] Gingras Y, Wallace ML (2010). Why it has become more difficult to predict Nobel Prize winners: A bibliometric analysis of nominees and winners of the chemistry and physics prizes (1901–2007). Scientometrics.

[CR30] Global research on coronavirus disease (COVID-19) (2020). https://www.who.int/emergencies/diseases/novel-coronavirus-2019/global-research-on-novel-coronavirus-2019-ncov

[CR31] Grogan, M. (2020, September 22). COVID-19 From A Time Series Perspective. *Medium*. https://towardsdatascience.com/covid-19-from-a-time-series-perspective-a5082903d836

[CR32] Huerta TR, Walker DM, Johnson T, Ford EW (2016). A time series analysis of cancer-related information seeking: Hints from the health information national trends survey (HINTS) 2003–2014. Journal of Health Communication.

[CR33] Incites Journal Citation Reports (2018). Category profile: Information science & library science (2018). https://jcr.clarivate.com/JCRCategoryProfileAction.action?year=2018&categoryName=INFORMATION%20SCIENCE%20%26%20LIBRARY%20SCIENCE&edition=SSCI&category=NU

[CR34] Iwami S, Mori J, Sakata I, Kajikawa Y (2014). Detection method of emerging leading papers using time transition. Scientometrics.

[CR35] Jiang F., Zhao, Z., & Shao, X. (2020). Time series analysis of COVID-19 infection curve: A change-point perspective. http://arxiv.org/abs/2007.04553 10.1016/j.jeconom.2020.07.039 PMC7392157 32836681

[CR36] Johnes G, Johnes J (1992). Apples and oranges: The aggregation problem in publication analysis. Scientometrics.

[CR37] Jones RH (1964). Spectral analysis and linear prediction of meteorological time series. Journal of Applied Meteorology.

[CR38] Kendall, G. (2015, October 15). The future of scientific publishing: Let’s make sure it’s fair as well as transparent. *The Conversation*. https://theconversation.com/the-future-of-scientific-publishing-lets-make-sure-its-fair-as-well-as-transparent-48900

[CR39] Kwon U, Geum Y (2020). Identification of promising inventions considering the quality of knowledge accumulation: A machine learning approach. Scientometrics.

[CR40] Larivière V, Sugimoto CR, Cronin B (2012). A bibliometric chronicling of library and information science’s first hundred years. Journal of the Association for Information Science and Technology.

[CR41] Leydesdorff L (1990). The prediction of science indicators using information theory. Scientometrics.

[CR42] Li X, Hitt LM (2008). Self selection and information role of online product reviews. Information Systems Research.

[CR43] Liu Y, Rousseau R (2008). Definitions of time series in citation analysis with special attention to the h-index. Journal of Informetrics.

[CR44] Luo X, Zhang J (2013). How do consumer buzz and traffic in social media marketing predict the value of the firm?. Journal of Management Information Systems.

[CR45] Ma R (2012). Discovering and analyzing the intellectual structure and its evolution of LIS in China, 1998–2007. Scientometrics.

[CR46] McClellan C, Ali MM, Mutter R, Kroutil L, Landwehr J (2017). Using social media to monitor mental health discussions—Evidence from Twitter. Journal of the American Medical Informatics Association.

[CR47] Merton RK (1968). Thematthew effect in science: The reward and communication systems of science are considered. Science.

[CR48] Montgomery DC, Jennings CL, Kulahci M (2008). Introduction to time series analysis and forecasting.

[CR49] Moya-Anegón F, Herrero-Solana V, Jiménez-Contreras E (2006). A connectionist and multivariate approach to science maps: The SOM, clustering and MDS applied to library and information science research. Journal of Information Science.

[CR50] Ni C, Sugimoto CR (2011). Four-facets study of scholarly communities: Artifact, producer, concept, and gatekeeper. Proceedings of the 2011 ASIS&T Annual Meeting.

[CR51] Niu N, Liu X, Jin H, Ye X, Liu Y, Li X, Chen Y, Li S (2017). Integrating multi-source big data to infer building functions. International Journal of Geographical Information Science.

[CR52] Organisation for Economic Co-operation and Development (OECD) (2020). Researchers (indicator). https://data.oecd.org/rd/researchers.htm

[CR53] *Overview—Health Information & Libraries Journal*. (2020). Wiley Online Library. https://doi.org/10.1111/(ISSN)1471-1842

[CR54] Perrote A, Ranganath R, Hirsch JS, Blei D, Elhadad N (2015). Risk prediction for chronic kidney disease progression using heterogeneous electronic health record data and time series analysis. Journal of the American Medical Informatics Association.

[CR55] Petersen AM, Penner O (2014). Inequality and cumulative advantage in science careers: A case study of high-impact journals. EPJ Data Science.

[CR56] Price DJ (1963). Little science, big science.

[CR57] Price DJ (1974). Science since Babylon.

[CR58] Priem J (2013). Beyond the paper. Nature.

[CR59] Rousseau R (1994). Double exponential models for first-citation processes. Scientometrics.

[CR60] Saboo AR, Kumar V, Park I (2016). Using big data to model time-varying effects for marketing resource (re)allocation. MIS Quarterly.

[CR61] Salgotra R, Gandomi M, Gandomi AH (2020). Time series analysis and forecast of the COVID-19 pandemic in india using genetic programming. Chaos, Solitons & Fractals.

[CR62] Shumway RH, Stoffer DS (2006). Time series analysis and its applications with R examples.

[CR63] Siegel, K. (2019, October 10). Can we predict which biologists are likely to win a Nobel Prize? *The Startup*. https://medium.com/swlh/can-we-predict-which-biologists-are-likely-to-win-a-nobel-prize-6a748e40e207

[CR64] Simkin M, Roychowdhury V, Cronin B, Sugimoto CR (2015). Do you sincerely want to be cited? Or Read before you cite. Scholarly Metrics Under the Microscope From Citation Analysis to Academic Auditing.

[CR65] Simpao AF, Ahumada LM, Desai BR, Bonafide CP, Gálvez JA, Rehman MA, Jawad AF, Palma KL, Shelov ED (2015). Optimization of drug-drug interaction alert rules in a pediatric hospital’s electronic health record system using a visual analytics dashboard. Journal of the American Medical Informatics Association.

[CR66] Small H (2006). Tracking and predicting growth areas in science. Scientometrics.

[CR67] Tahamtan I, Afshar AS, Ahamdzadeh K (2016). Factors affecting number of citations: A comprehensive review of the literature. Scientometrics.

[CR68] The World Bank (2018). Research and development expenditure (% of GDP). *UNESCO Institute for Statistics*. https://data.worldbank.org/indicator/GB.XPD.RSDV.GD.ZS?view=chart

[CR69] Tonta Y (2018). Does monetary support increase the number of scientific papers? An interrupted time series analysis. Journal of Data and Information Science.

[CR70] Tripathy P, Tripathy PK (2017). Fundamentals of research: A dissective view.

[CR71] Tseng Y-H, Tsay M-Y (2013). Journal clustering of library and information science for subfield delineation using the bibliometric analysis toolkit: CATAR. Scientometrics.

[CR72] ULRICHSWEB Global Serials Directory. (2020). https://ulrichsweb.serialssolutions.com

[CR73] Waldrop, M. M. (2008, May). *Science 2.0—Is open access science the future?* https://www.scientificamerican.com/article/science-2-point-0/

[CR74] Walters GD (2006). Predicting subsequent citations to articles published in twelve crime-psychology journals: Author impact versus journal impact. Scientometrics.

[CR75] White HD, McCain KW (1998). Visualizing a discipline: An author co-citation analysis of information science, 1972–1995. Journal of the American Society for Information Science.

[CR76] Wu QW, Zhang C, Hong Q, Chen L (2014). Topic evolution based on LDA and HMM and its application in stem cell research. Journal of Information Science.

[CR77] Xie Y (2014). ‘Undemocracy’: Inequalities in science. Science.

[CR78] Ye FY, Rousseau R (2008). The power law model and total career h-index sequences. Journal of Informetrics.

[CR79] You H, Li M, Hipel KW, Jiang J, Ge B, Duan H (2017). Development trend forecasting for coherent light generator technology based on patent citation network analysis. Scientometrics.

[CR80] Zeisset PT (1998). Disseminating economic census data. Government Information Quarterly.

[CR81] Zhang T (2020). Will the increase in publication volumes “dilute” prestigious journals’ impact factors? A trend analysis of the FT50 journals. Scientometrics.

[CR82] Zhang Y, Shah D, Foley J, Abhishek A, Lukito J, Suk J, Kim SJ, Sun Z, Pevehouse J, Garlough C (2019). Whose lives matter? Mass shootings and social media discourses of sympathy and policy, 2012–2014. Journal of Computer-Mediated Communication.

